# Assessment of central venous catheterization in a simulated model using a motion-tracking device: an experimental validation study

**DOI:** 10.1186/s13022-016-0025-6

**Published:** 2016-02-12

**Authors:** Julián Varas, Pablo Achurra, Felipe León, Richard Castillo, Natalia De La Fuente, Rajesh Aggarwal, Leticia Clede, María P. Bravo, Marcia Corvetto, Rodrigo Montaña

**Affiliations:** Experimental Surgery and Simulation Center, Department of Digestive Surgery, Clinic Hospital, School of Medicine, Pontificia Universidad Católica de Chile, Santiago, Chile; Anesthesiology Division, Clinic Hospital, School of Medicine, Pontificia Universidad Católica de Chile, Marcoleta 367, Santiago, Chile; Department of Surgery, Faculty of Medicine, McGill University, Montreal, Canada; Arnold and Blema Steinberg Medical Simulation Centre, Faculty of Medicine, McGill University, Montreal, Canada

**Keywords:** Medical simulation, Central venous catheterization, Tracking motion device, Objective skills assessment, Medical training

## Abstract

**Background:**

Central venous catheterization (CVC) is a basic requirement for many medical specialties. Simulated training in CVC may allow the acquisition of this competency but few reports have established a valid methodology for learning and acquiring procedural skills for CVC. This study aims to validate the use of a tracking motion device, the imperial college surgical assessment device (ICSAD), by comparing it with validated global rating scales (GRS) to measure CVC performance in a simulated torso.

**Methods:**

Senior year medical students, first and last year residents (PGY1, LYR), and expert anesthesiologists performed a jugular CVC assessment in a simulated model (Laerdal IV Torso). A validated GRS for objective assessment of technical skills and motion analysis by ICSAD was used. Statistical analysis was performed through Mann–Whitney and Kruskal–Wallis tests for construct validity and Spearman correlation coefficients between the ICSAD and GRS scores for concurrent validity between both.

**Results:**

32 subjects were recruited (10 medical students, 8 PGY1, 8 LYR and 8 experts). Total path length measured with ICSAD and GRS scores were significantly different between all groups, except for LYR compared to experts (p = 0.664 for GRS and p = 0.72 for ICSAD). Regarding jugular CVC procedural time, LYR and experts were faster than PGY1 and MS (p < 0.05). Spearman correlation coefficient was −0.684 (p < 0.001) between ICSAD and GRS scores.

**Conclusions:**

ICSAD is a valid tool for assessment of jugular CVC since it differentiates between expert and novice subjects, and correlates with a validated GRS for jugular CVC in a simulated torso.

## Background

Central venous catheterization (CVC) is an essential competency required for many medical specialties [1, 2]. Annually, 5 million CVC are performed only in the United States [3], with serious and life-threatening complications occurring in up to 5–26 % [4] of the cases. These adverse events are inversely related to practitioner’s clinical experience [5].

Nowadays, simulated training for acquiring technical skills is becoming widespread for many medical specialties [2, 6, 7], from simple procedures like a venous puncture to more complex surgical procedures like a laparoscopic jejuno-jejunostomy [8–10], shortening the learning curves of residents while doing so in a safe and controlled environment [11]. Few studies have evaluated the acquisition of CVC proficiency through simulated models and their educational effectiveness [11–15]. These studies have shown an increase in the rate of successful CVC [16] and a decrease of associated complications after the simulated training programs [17, 18].

The Accreditation Council for Graduate Medical Education (ACGME) recommends the use of simulation and checklists as the “most desirable” evaluation methods for the assessment of competency in procedural skills [13], and they have been commonly used for the evaluation of CVC [4, 16, 19, 20]. However structured global rating scales as the objective structured assessment of technical skill (OSATS) [21, 22] have demonstrated better assessment and discrimination of different levels of skills than checklists previously used [13].

The imperial college surgical assessment device (ICSAD) is a device that tracks hand-motion of the operator during a procedure, using sensors placed on the back of the trainee’s hands. Total path length of both hands is registered, providing an effective index of technical skill during a procedure [7, 23]. The ICSAD has demonstrated construct validity in many surgical procedures [23, 24] and it has been used for objective assessment of proficiency in anesthetic procedures such as labor epidural placement [25] and ultrasound-guided peripheral nerve blockade [26]. To our knowledge, there are no previous reports using motion-tracking devices to assess proficiency in CVC.

The validation of a motion-tracking device may complement the use of global rating scales (OSATS) in assessing better the differences between expert and novices procedural skills.

Therefore, the aim of this study is to establish the construct and concurrent validity of the tracking motion device (ICSAD) in assessing CVC in a simulated model.

## Methods

The Institutional Review Board approved the study, and written informed consent was obtained from all participants.

**Fig. 1:**
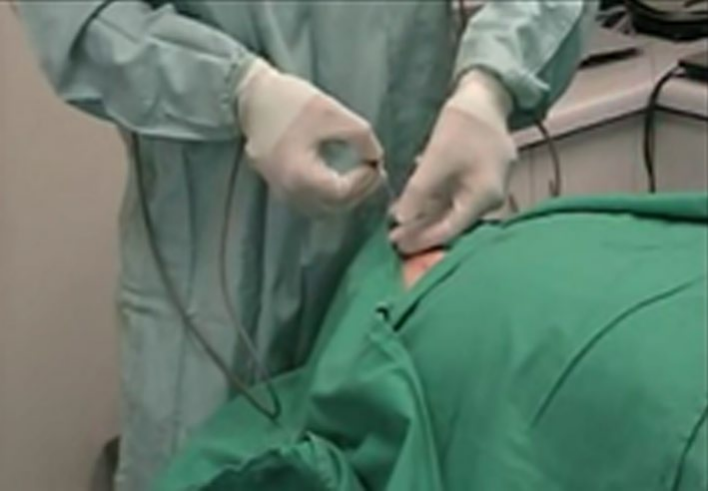
Simulated jugular central venous catheterization assessment in an Adult IV simulated model, using a tracking motion device on their hands: the ICSAD

Four different groups from Pontificia Universidad Católica de Chile Medical School were studied between November–December 2013. All students and postgraduate residents that were available for the period of assessment were recruited. Based on previous studies by our group a sample study of eight participants per group was calculated. All groups underwent a simulated jugular central venous catheterization assessment in an Adult IV simulated model, using tracking motion sensors attached on their hands (ICSAD) as shown in Fig. 1 [23, 24].

Residents keep an active registration of the number of procedures they complete during the 3 year residency. Prior jugular CVC experience of the different groups is shown in Table 1.

**Table 1:** Number of prior CVC insertion by each group evaluated

	Students (n = 10)	PGY1 (n = 8)	LYR (n = 8)	Experts (n = 8)
Median (range)	0 (0–1)	4 (0–20)	50 (15–80)	>100 (200–400)

*CVC* Central venous catheterization, *PGY1* first year post graduate residents, *LYR* last year residents

### Prior to assessment

Before assessment, all groups were gathered at a 2 h master class where they were explained how to perform a jugular CVC in the simulated model. In addition, a DVD video was provided to each student with a step-by-step instructional guide emphasizing the key issues related to the procedure and most common mistakes.

### Jugular CVC assessment

After the introductory class, all groups were assessed performing one jugular CVC in an Adult Laerdal IV bench model (Laerdal IV Torso; Laerdal Medical Corp, Wappingers Falls, NY) [13, 27]. Each task was video-recorded and then blindly assessed by three independent expert anesthesiologists using a validated OSATS global rating scale (Modified from Ma et al. [13]). Economy of movements was assessed using the ICSAD from the beginning of the technical procedure (total path length was measured in meters). Procedure time (in seconds) was also recorded. Inter-rater reliability between OSATS evaluators was calculated using Kappa coefficient (0–1) [28, 29].

### Statistical analysis

Data was analyzed with the Statistical Package for the Social Sciences version 15.0 (SPSS, Chicago, IL, USA) using non-parametric tests. Mann–Whitney and Kruskal–Wallis tests were used for each variable and the results were exposed in median (range).

Spearman correlation coefficients between the ICSAD scores and the validated OSATS global rating scale scores were calculated to establish the concurrent validity of ICSAD [13]. Following the Cohen guidelines, a positive or negative value between 0.5 and 1.0 indicates a large effect, 0.3–0.5 indicates a medium effect, and 0.3–0.1 indicates a small effect [30]. P value was considered statistically significant when <0.05.

## Results

A total of 32 subjects were recruited, divided in 10 medical students (MS), 8 PGY1, 8 LYR and 8 expert anesthesiologist. Inter-rater reliability was established between the three OSATS evaluators, obtaining a Kappa coefficient of 0.76 (CI 0.58–0.92).

**Fig. 2:**
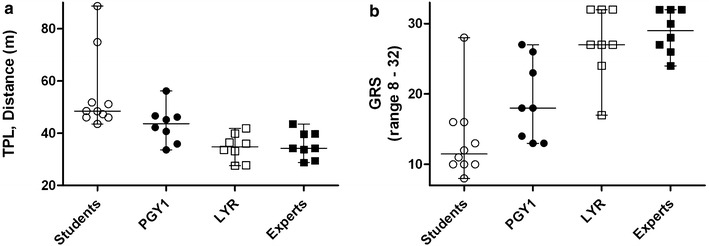
Comparison between final year medical students, PGY1, LYR and expert in total path length (TPL) (**a**) and global rating scale (GRS) scores (**b**)

Results of total path length measured with ICSAD, OSATS global rating scores and procedural time are shown in Table 2. Regarding ICSAD total path length, all groups had significant differences between them, except for LYR compared to experts (p = 0.664; Fig. 2a).

**Table 2:** JCVC assessment (bench model)

	Students (n = 10)^A^	PGY1 (n = 8)^B^	LYR (n = 8)^C^	Experts (n = 8)^D^	p value^AB^	p value^BC^	p value^BD^	p value^CD^
GRS (8–32)	11.5 (8–28)	18 (13–27)	27 (17–32)	29 (24–32)	0.029	0.014	0.004	0.664
TPL (m)	48.5 (44–89)	43 (33–56)	35 (28–42)	34 (28–44)	0.028	0.015	0.04	0.72
Procedural time (s)	344 (218–609)	243 (121–571)	133 (111–339)	122 (116–201)	0.172	0.053	0.015	0.694

Comparison between final year medical students, PGY1 and LYR anesthesiology residents, and expert Anesthesiologists

*GRS* Global rating scores, *TPL* total path length, *CVC* central venous catheterization, *PGY1* first year postgraduate residents, *LYR* last year residents

^AB^p values obtained when comparing columns ^A^ and ^B^ with Mann–Whitney test

^BC^p values obtained when comparing columns ^B^ and ^C^ with Mann–Whitney test

^BD^p values obtained when comparing columns ^B^ and ^D^ with Mann–Whitney test

^CD^p values obtained when comparing columns ^C^ and ^D^ with Mann–Whitney test

In OSATS median scores, there were significant differences between all groups, except for LYR compared to experts (p = 0.72; Fig. 2b).

Finally, concerning procedural time, there were no differences between MS compared to PGY1 (p = 0.172) and LYR compared to experts (p = 0.694). However, the last two had significant less procedural time than PGY1 and MS (p = 0.015).

Spearman correlation coefficient between the total path length measured with ICSAD and the validated OSATS global rating scale score demonstrated a strong correlation, with a Spearman correlation coefficient of −0.684 (p < 0.001).

## Discussion

Central venous catheterization is commonly learned during residences trough the “see one, do one, teach one” approach [31]. This apprenticeship model requires that inexperienced residents perform the procedure on patients, in a clinical setting with few or none standardized methodology to teach or evaluate the procedure [31]. Therefore, this learning model does not ensure proficiency in practical skills and jeopardizes patients’ safety [32].

Surgical specialties have vast experience in objective assessment of technical skills for a procedure [7]. Global rating scales, specific checklists and motion analysis like ICSAD have been used to evaluate many surgical procedures, establishing a good correlation between scores obtained and surgeon’s competency level [7, 23, 24]. In the case of anesthesia, only few reports have used objective assessment of technical skills, demonstrating a good correlation between scores obtained with the assessment tools and the expertise level [19, 20]. Tracking motion devices like ICSAD have demonstrated construct validity in discriminating the different levels of expertise in anesthetic procedures, such as an epidural catheter insertion [25] or an ultrasound-guided peripheral nerve blockade [26].

This is the first validation study reporting the use of ICSAD as an assessment tool for jugular CVC. The exercise of this motion device in the evaluation of jugular CVC allows the obtainment of quantitative data that complements global rating scales for differentiating between novice and expert, thus, adding construct validity to the simulated model. Both of these tools were more useful in discriminating the level of expertise when compared to procedural time as an assessment measurement (Table 2).

In the case of concurrent validity of ICSAD for jugular CVC, a good correlation was achieved with the previous validated OSATS global rating scale. The ICSAD is an objective numeric tool, non-dependent of the evaluating teacher which reduces assessment bias.

No significant differences were found between experienced residents (LYR) and expert anesthesiologists in terms of total path length measured by ICSAD, global rating scale or time of procedure. A possible explanation for this result is that most LYR residents have already flattened their learning curves and achieved the minimum proficiency needed to perform this procedure. They are considered experienced non-experts and perform well on routine problems by unreflectively and automatically applying the standard technique [33]. Hayter et al. [25] had similar findings when they assessed the epidural catheter insertion in residents, and proposed to add variables such as non-standard patient scenarios in order to discriminate in a more subtle way the expertise level [25].

Our main study limitation is small sample groups, mainly due to local difficulties in gathering residents for an experimental protocol in our institution. However, there were statistically significant differences between the assessed groups when comparing the tracking motion measures; concluding that ICSAD may help differentiate between different skills level in CVC simulated assessment.

Finally, this is the first report establishing construct validity of the Laerdal IV Torso model. This training bench model, with ICSAD and GRS used as assessing tools, allows to discriminate between different levels of expertise. The technical skills gap observed in this simulated model between novices and experts provides learning opportunities for trainees, setting the cutoff scores to be achieved. Further predictive validity studies are needed in order to determine whether the skills acquired through the simulated training may or not transfer to real life scenario with patients.

In conclusion, ICSAD was correctly validated for assessing jugular CVC in a simulated model, as it discriminates between expert and novices and correlates with validated OSATS global rating scale. To have as many as possible instruments for evaluating procedural skills such a jugular CVC may improve the objectification of competency acquisition in real patients.

## Abbreviations

CVC: central venous catheterization
GRS: global rating scales
ICSAD: imperial college surgical assessment device
LYR: last year residents
MS: medical students
OSATS: objective structured assessment of technical skill
PGY1: post-graduate year 1
